# Efficient Elimination of Inhaled Nanoparticles from the Alveolar Region: Evidence for Interstitial Uptake and Subsequent Reentrainment onto Airways Epithelium

**DOI:** 10.1289/ehp.9685

**Published:** 2007-02-06

**Authors:** Manuela Semmler-Behnke, Shinji Takenaka, Steffanie Fertsch, Alexander Wenk, Jürgen Seitz, Paula Mayer, Günter Oberdörster, Wolfgang G. Kreyling

**Affiliations:** 1 GSF - National Research Center for Environment and Health, Institute of Inhalation Biology, Neuherberg/Munich, Germany; 2 Inamed Research GmbH & Co. KG, Gauting, Germany; 3 Department of Environmental Medicine, University of Rochester, Rochester, New York, USA; 4 GSF - National Research Center for Environment and Health, Institute of Inhalation Biology and Focus Network Aerosols and Health, Neuherberg/Munich, Germany

**Keywords:** alveolar macrophages, bronchoalveolar lavage, clearance, inhalation, nanoparticles, reentrainment, relocation, retention, translocation

## Abstract

**Background:**

There is ongoing discussion that inhaled nanoparticles (NPs, < 100 nm) may translocate from epithelial deposition sites of the lungs to systemic circulation.

**Objectives and Methods:**

We studied the disappearance of NPs from the epithelium by sequential lung retention and clearance and bronchoalveolar lavage (BAL) measurements in healthy adult Wistar Kyoto (WKY) rats at various times over 6 months after administration of a single 60- to 100-min intratracheal inhalation of iridium-192 (^192^Ir)–radiolabeled NPs. A complete ^192^Ir balance of all organs, tissues, excretion, remaining carcass, and BAL was performed at each time point.

**Results:**

Directly after inhalation we found free NPs in the BAL; later, NPs were predominantly associated with alveolar macropages (AMs). After 3 weeks, lavageable NP fractions decreased to 0.06 of the actual NP lung burden. This is in stark contrast to the AM-associated fraction of micron-sized particles reported in the literature. These particles remained constant at about 0.8 throughout a 6-month period. Three weeks after inhalation, 80% of the retained Ir NPs was translocated into epithelium and interstitium.

**Conclusion:**

There is a strong size-selective difference in particle immobilization. Furthermore, AM-mediated NP transport to the larynx originates not only from the NP fraction retained on the epithelium but also from NPs being reentrained from the interstitium to the luminal side of epithelium. We conclude that NPs are much less phagocytized by AMs than large particles but are effectively removed from the lung surface into the interstitium. Even from these interstitial sites, they undergo AM-mediated long-term NP clearance to the larynx.

Recent epidemiologic studies provide evidence that an increase in atmospheric nanoparticles (NPs) is associated with adverse cardiovascular effects in susceptible parts of populations (Ibald-Mulli et al. 2002; Wichmann and Peters 2000; Wichmann et al. 2000) such as the elderly and people with underlying diseases of various origins [U.S. Environmental Protection Agency (U.S. EPA) 2004]. Clearance pathways of inhaled nanoparticles (NPs) from the lungs are assumed to differ from those of larger particles (> 100 nm diameter). In other words, lung retention and clearance of NPs are thought to be mediated less by alveolar macrophages (AMs) than are those of larger particles because AMs *in vivo* are not efficient in phagocytizing NPs—as they do in particles > 100 nm (Kreyling and Scheuch 2000). However, Semmler et al. (2004) found no difference between pulmonary clearance kinetics of NPs and that of larger particles, suggesting that underlying clearance mechanisms for different-sized particles ranging from nano-sized to micron-sized might be the same. This is puzzling given the discrepancy between the very efficient phagocytosis of micron-sized particles by AMs on the one hand and the very inefficient uptake of NPs by these cells on the other. How can these opposite initial events of AM–particle interaction lead to the same pulmonary clearance kinetics for both particle types?

We hypothesize that the answer lies in the well-described propensity of NPs deposited in the lungs to translocate to epithelial cells (ECs) and interstitium and, as suggested more than 30 years ago by Brundelet (1965) and Tucker et al. (1973), are carried via the lymph flow to bronchial and bronchiolar sites, including bronchial-associated lymphatic tissue (BALT), where they are excreted again into the airway lumen. Consistent with this hypothesis, Ferin and Oberdörster (1992), based on results from a rat inhalation study with ultrafine (< 100 nm) titanium dioxide (TiO_2_), reported histologic evidence for the existence of two distinct clearance pathways for particles in the peripheral lung via *a*) airways and *(b*) the interstitial lymphatics and back to the airways, and that these are interconnected. However, these authors also cautioned that “it is difficult to prove direction of a clearance pathway from a static histological observation,” which was the only evidence they had. Thus, our aim in the present study was to determine the clearance dynamics of inhaled NPs retained in different compartments of the rat’s lower respiratory tract over a 6-month period, with special attention to the association of NPs with AMs.

## Material and Methods

### Approach

A common approach to investigating the clearance kinetics of particles in the lungs is the use of poorly soluble particles labeled with a convenient radioactive gamma emitter. We found iridium-192 (^192^Ir) NPs to be the appropriate material for studying clearance kinetics because of the very low solubility of Ir, even as an NP (Kreyling and Scheuch 2000; Semmler et al. 2004), although we are aware that Ir is not a relevant component of the ambient aerosol. In a previous study by Kreyling et al. (2002), the authors found that ultrafine poorly soluble ^192^Ir particles were predominantly retained in the lungs during the first week after inhalation. The NPs deposited in thoracic airways and lung periphery were cleared predominantly via airways and larynx into the gastrointestinal tract (GIT) and feces. Our goal in the present study was to determine the long-term alveolar retention of these NPs and to compare our results with those of Lehnert et al. (1989) for 2-μm particles by analyzing retention kinetics in different compartments of the lung. In the study by Lehnert et al., the lavageable fraction of 2-μm-sized instilled polystyrene particles was quantitatively distinguished from the nonlavageable fraction at various times during 6 months, which indicated that the vast amount of particles stay at all times on the lung epithelium; this was achieved by dissolving tissue, then counting the fluorescent particles. In a similar study in Syrian golden hamsters, Ellender et al. (1992) obtained similar results after the hamsters inhaled micron-sized glass particles.

### Animals

Healthy, male WKY rats (WKY/Kyo@Rj rats, Janvier, Le Genest Saint Isle, France) 8–10 weeks of age and approximately 250 g body weight (bw) were housed in pairs in a humidity-controlled (55% relative humidity) and temperature-controlled (22°C) room in individually ventilated cages (VentiRack, cage type CU-31; BioZone Limited, Margate, Kent, UK), maintained on a 12-hr day/night cycle. Rodent diet and water were provided *ad libitum*. All animals used in this study were treated humanely and with regard for alleviation of suffering. The studies were conducted under federal guidelines for the use and care of laboratory animals and were approved by the District of Upper Bavaria, Approval No. 211-2531-108/99 and by the GSF Institutional Animal Care and Use Committee.

### Aerosol production and characterization

The ultrafine ^192^Ir aerosol was produced using a spark generator as described previously (Kreyling et al. 2002). The spark frequency was 3 Hz in an argon stream of 3 L/min; immediately after condensational particle formation, the aerosol was neutralized by an electric charge with a radioactive krypton-85 source. The aerosol then was diluted with nitrogen and adjusted to 20% oxygen, 50–60% relative humidity, and 37°C. This created a log normal distribution of aerosol with a concentration of 1–3 × 10^7^ cm^−3^ [continuously monitored by a condensation particle counter (CPC 3022A; TSI GmbH, Aachen, Germany) in a controlled diluted aerosol sample], a count median diameter (CMD) between 17–20 nm, and a geometric standard deviation of 1.6 [continuously determined with a differential particle mobility analyser (Classifier 3071 + CPC 3010; TSI GmbH)]. Specific Ir activity was 10 GBq/g at a reference date obtained by neutron activation of the Ir electrodes of the spark generator in a research reactor (Hahn-Meitner-Institute, Berlin, Germany). Mass concentration was calculated as 0.7 mg/m from the radioactivity measurement of an integral filter sample and the sampled aerosol volume.

### Inhalation

To focus the analysis on the kinetics of particle clearance from the lungs unaffected by external contamination, we avoided pelt contamination and particle deposition in extrathoracic airways by using our previously developed method of intratracheal intubation inhalation.

In preparation for the exposure, WKY rats (32 in total, 4 at a time) were anaesthetized with an intramuscular injection of a mixture of medetomidine (15 μg/100 g bw), midazolam (0.2 mg/100 g bw), and fentanyl (0.5 μg/100 g bw). For endotracheal intubation, a flexible cannula (16 ga, 2 inches in length) was placed in the upper trachea under visual control and sealed against outside air with a modified pipette tip around the catheter wedged gently into the laryngeal opening (Kreyling et al. 1993, 2002; Osier and Oberdörster 1997). Each animal was placed in a self-made plethysmograph and connected with its endotracheal cannula to the aerosol line outside of the plethysmograph. The plethysmographs were temperature controlled at 37°C. Ventilation was computer controlled with a negative pressure of −1.5 kPa applied to the plethysmograph for 1 sec of inspiration followed by 0.5 sec allowing spontaneous expiration to functional residual capacity (FRC) at ambient air pressure; the resulting breathing frequency was 40 breaths/min. This ventilation pattern caused inspiration from FRC to 75–80% of total lung capacity of the rat; therefore, animals were slightly hyperventilated and did not breathe spontaneously but followed the computer-controlled breathing pattern. For the purpose of radiation protection, the entire aerosol system was *a*) maintained at −30 Pa low pressure on average and *b*) installed in a glove box, which was ventilated continuously through an absolute particle filter and maintained at −5 to −10 Pa below ambient air pressure in the laboratory.

After 60–100 min of inhalation, depending on the anticipated deposited NP dose, rats were removed from their plethysmographs, extubated, and received an anesthesia antidote by a subcutaneous injection of a mixture of atipa-mezol (0.075 mg/100 g bw), flumazenil (20 μg/ 100 g bw), and naloxone (10 μg/100 g bw).

### Clearance and retention: NP clearance

After exposure, rats were maintained in metabolic cages for the first 7 days, then singly in individually ventilated cages. Excreta were collected quantitatively throughout the study; during the first week daily urine and feces samples were collected separately in the metabolic cages; later urine and feces samples were collected together with the cage bedding for periods of 3 days and up to 7 days. Fecal droppings were separated from bedding to allow for distinction of urinary excretion in bedding from fecal excretion in droppings.

### Deposition and terminal retention

At 0, 6, and 24 hr, 3 and 7 days, 3 weeks, and 2 and 6 months after exposure, four rats each were killed by exsanguinations after ip anesthesia with ketamine (10 mg/100 g bw) and xylazine (0.5 mg/100 g bw). Completely balanced retention of ^192^Ir activity retained in the body was quantified by gamma spectroscopy in a 1-L well-type scintillation detector (Eurisys/ Canberra, Rüsselsheim, Germany) corrected for background radiation and detector geometry and sensitivity. The total excreted fraction throughout the entire study was also determined gamma spectroscopically. Hence, total ^192^Ir activity of retention plus excretion represents the initially deposited ^192^Ir activity.

### Gamma camera image of ^192^Ir NP distribution in the lungs

Excised lungs of rats killed immediately after inhalation were inflated to 3.5 kPa corresponding to total lung capacity and air dried. The dried lungs were subsequently placed under a single photon emission computed tomograph gamma camera (SPECT) (Prism 2000; Philips Medizin Systeme GmbH, Hamburg, Germany) equipped with a pinhole collimator and adjusted for gamma energy of 310 keV of ^192^Ir; images were collected for 24 hr. The planar image of the activity distribution was compared with digital photographs resembling the same optical geometry for qualitative checking of homogeneous ^192^Ir activity distribution throughout the lungs and their periphery.

### Bronchoalveolar lavage (BAL)

At the above-mentioned time points, BAL was performed. After perforating the diaphragm, 5 mL phosphate-buffered saline (PBS) without Ca^2+^ and Mg^2+^ was administered 6 times into the lungs *in situ* under gentle massage of the thorax and recovered. The recovered BAL fluid (about 90% of instilled saline) was centrifuged at 500 g, for 20 min at 20°C, and lavaged cells were separated from supernatant. The total number of lavaged cells was counted with a hemocytometer by a dilute of the spun-down cells. Viable cells were distinguished by exclusion of trypan-blue staining. Free NPs unattached to BAL cells were recovered in the supernatant of the recovered BAL fluid. Lavaged cell numbers and cell viability were determined using a hemocytometer. Cyto-centrifuged slides of spun-down lavaged cells were prepared for each animal and stained with Diff-Quik (Dade Behring, Newark, DE, USA) for cell differential counts. The ^192^Ir activity within the lavaged cells and in the supernatant was quantified by gamma spectroscopy in a 1-L well-type scintillation detector.

### Lavageable fraction of NPs in WKY rats

Because of the very low solubility of the ^192^Ir particles, the measured ^192^Ir activity in both fractions of BAL is particle associated, and the dissolved fractions are negligible. In addition, we proved that free ultrafine ^192^Ir particles in saline were not significantly (< 2%) spun down under the conditions of centrifugation chosen. Hence, ^192^Ir radioactivity determined in the lavaged cell pellets represents ^192^Ir particles either phagozytized by or adherent to AMs, whereas the activities in the supernatants represent free ^192^Ir particles in the BAL fluid.

### Data analysis

Retention and excretion data were normalized and expressed as fractions of the initial lung burden. In addition, fractional rates of lavaged and excreted ^192^Ir NPs were determined as fractions of the contemporary lung burden at given time points when indicated.

All radioactivity data were background-corrected and calculated for the date of neutron activation.

## Results

### Deposition

Deposited ^192^Ir doses of the exposed rats at the various time points were shown previously (Semmler et al. 2004). Recalculation of these data yielded a mean-deposited Ir mass of 2 μg/rat during the 1-hr exposure with the exemption of the 6-month study in which we increased the exposure time by a factor of 1.7, resulting in an increased deposited Ir mass of 3.5 μg/rat.

As shown earlier, the ultrafine ^192^Ir-labeled particles were virtually insoluble (< 1% of Ir mass within 7 days of incubation in PBS). Therefore, radioactivity measurement is proportional to the mass of the ultra-fine Ir particles.

### Particle distribution in the lungs

**Figure 1:**
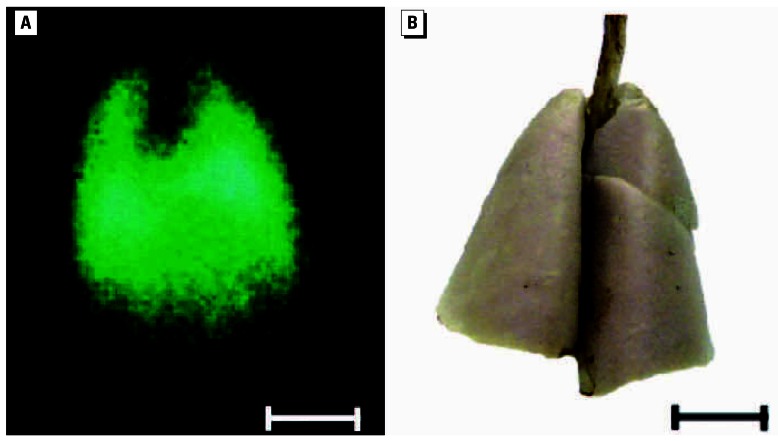
(*A*) SPECT gamma camera (Prism 2000; Philips) image of ^192^Ir activity distribution in the dried lungs applying a pinhole collimation geometry. (*B*) Air-dried rat lungs, immediately removed after inhalation and air dried at total lung capacity, dorsal view; bar = 10 mm.

Distribution patterns of inhaled ^192^Ir NPs in the rat lungs were acquired with SPECT gamma camera images. These images showed radioactivity throughout the rat lungs after inhalation of ^192^Ir NPs by the intubated and ventilated rats (Figure 1). Note, however, the resolution of the gamma camera does not allow distinguishing between alveolar region and bronchial region of the rat lungs.

### Particle retention and clearance

Depostion and long-term particle retention and clearance kinetics were reported in detail recently (Semmler et al. 2004).

### Bronchoalveolar lavage (BAL)

**Figure 2:**
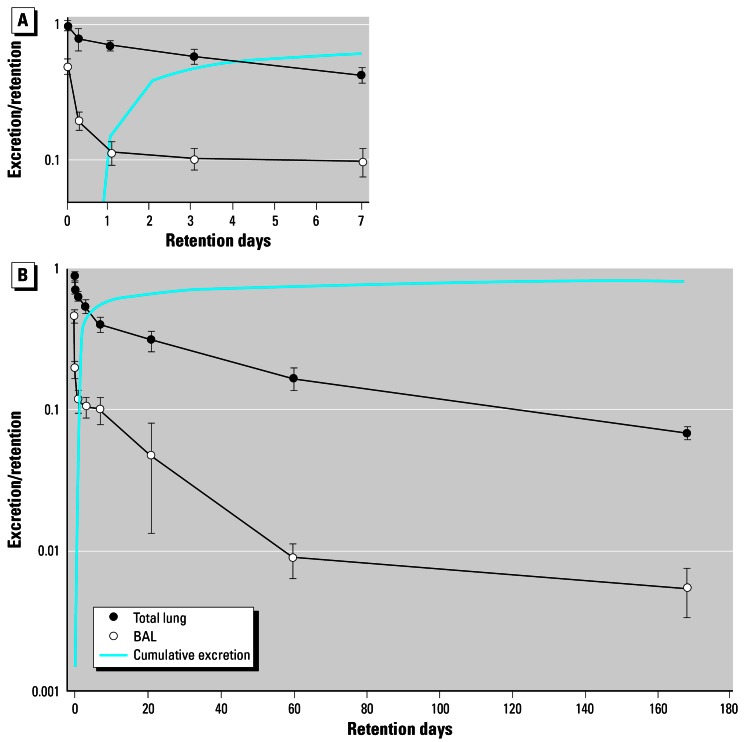
(*A*) Lung retention, cumulative fecal excretion, and lavaged fraction of ^192^Ir NPs with 17- to 20-nm median diameter during 6 months after a single 1- to 1.5-hr inhalation. (*B*) Expands the data of the first week after inhalation. Total lung retention data are the sum of measured lung and BAL fraction from each animal. All fractions are normalized to the initially deposited ^192^Ir NPs in the lungs. At each time point, data obtained from four rats are averaged and error bars indicate SD.

Figure 2 compares the retention of total Ir NPs in the lungs with the lavaged fractions throughout the 6-month period; fractions are normalized to the initially deposited lung burden. During the first 2 months, the recovered NP fraction drops much faster in BAL than in lungs. Thereafter, BAL and lung fractions run parallel.

Data for NPs accessible to BAL were reported recently (Semmler et al. 2004) and are shown in Table 1. The fraction of polymorphonuclear leukocytes (PMNs) showed a moderate increase immediately after the 1-hr ventilation of intubated rats. The elevated fraction persisted for 24-hr. Three days and longer after inhalation, PMNs of the BAL cell count had decreased to a physiologic control level of about 0.7 %; therefore, this transient and minor inflammation is not expected to affect the long-term clearance of the inhaled NPs. Because similar elevated fractions of PMNs have been observed 1 day after clean air intra-tracheal intubation inhalation, the ventilation via the endotracheal tube appears to be the cause of this transient inflammatory reaction.

The average number of lavaged cells over all individual BALs of Ir NP–exposed rats was 3.7 ± 3.13 × 10^6^. The high cell count at 21 days after inhalation is unexplained, as there was no observed adverse response.

#### Free NP fraction in BAL

**Figure 3:**
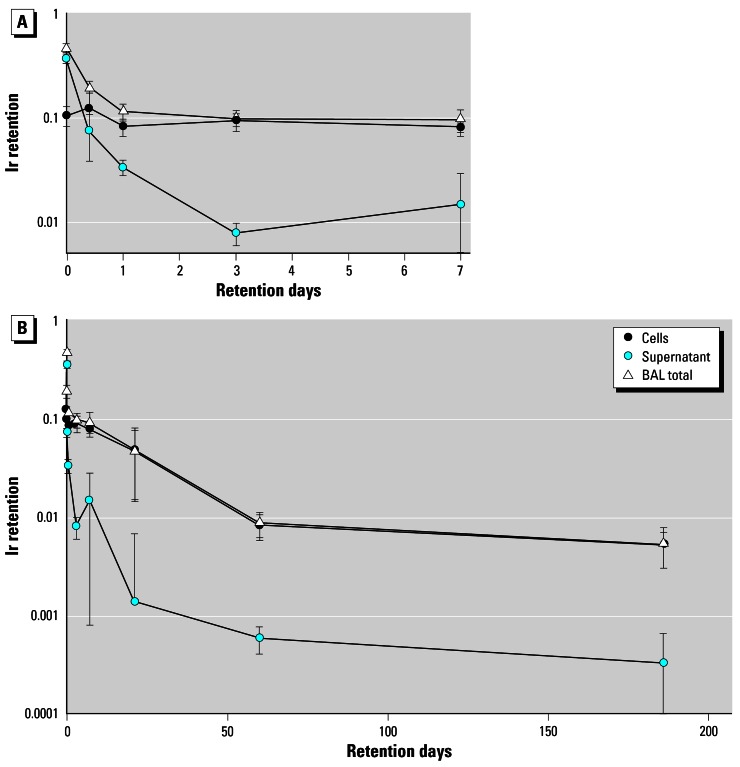
(*A,B*) ^192^Ir NP fraction of lung deposit as determined from ^192^Ir radioactivity measurements in cells and supernatant after separation by centrifugation of the BAL at different time points. (*B*) Expands the data of the first week after inhalation. All fractions are normalized to the initially deposited ^192^Ir NP in the lungs. At each time point, data obtained from four rats are averaged and error bars indicate SD.

Immediately after ^192^Ir NP inhalation, a fraction (NP_lav_) of 0.46 of the deposited NP was recovered by BAL (Table 1). Of this NP_lav_, only an NP fraction of 0.22 ± 0.05 was in the cell pellet (NP_CP_) and 0.78 were free NPs in the supernatant. The NP_CP_ fraction in the cell pellet increased rapidly on the first day. At day 3 only about 5–10% of the lavaged NPs were free in the BAL fluid. This is in agreement with studies using micron-sized particles that reported negligible fractions of free particles even 6 hr after inhalation (Brain 1985). In Figure 3 the lavaged NP fractions and those of free NPs compared with AM-associated NPs are plotted relative to the initial lung deposit, thereby indicating a sharp decrease of the free NPs, in particular; the free NP fraction dropped rapidly to about 1% during the first week and < 0.1% later.

## Discussion

The ^192^Ir particles appeared to be suitable for these studies because of their very low *in vitro* solubility and their simple biokinetics shown in previous investigations (Kreyling et al. 2002; Semmler et al. 2004). Administering the aerosol via an endotracheal tube allowed thoracic particle deposition and quantitatively balanced clearance measurements to begin immediately after inhalation, as no particles deposited in extrathoracic airways or contaminated the pelt. It is important to consider that the 1-hr procedure of intubated ventilation is likely to initiate a transient inflammation during the first hours after inhalation.

Ultrafine Ir NPs, 17–20 nm, were retained mainly in the lungs. At any time, NPs were cleared predominantly from the peripheral lung via the airways into the GIT and were found in feces (Semmler et al. 2004). Fecal excretion throughout the entire study of retention confirmed the predominance of this clearance pathway as shown previously.

Only directly after and 6 hr after Ir particle inhalation, was an appreciable amount of free particles found in the BAL fluid. At later times this fraction almost diminished. As a result, nearly all Ir NPs were associated with AMs in the BAL fluid. Interestingly this lavageable NP fraction declined to < 0.2 of the totally retained NPs over time. These fractions were much lower than the 0.8 fractions reported in studies using micron-sized particles (Ellender et al. 1992; Lehnert et al. 1989). In contrast, the daily cleared NP fractions in larynx and fecal excretions were similar to those measured for micron-sized particles as dicussed earlier (Semmler et al. 2004). To better understand the biokinetic fate of NPs while being retained in the lungs, we performed an additional analysis and comparison with existing data for micron-sized particles. These yielded three results, some of which had been hypothesized in the past:

- The major fraction of Ir NPs disappeared from the epithelial surface and were relocated within the epithelium and in the inter-stitium (Oberdörster et al. 2000, 2005).
- Surprisingly, from this interstitial NP fraction, Ir NPs continued to reentrain back onto the lung epithelium adding to the macrophage-mediated clearance transport to the larynx and fecal excretions (Ferin and Oberdörster 1992; Green 1973).
- Ir NP translocation into circulation was measurable as we showed previously, but the fraction was much less than that re-entraining back onto the epithelium.

### Determination of total AM population in WKY rats

Lehnert et al. (1989) used an “exhaustive” lavage technique that enabled recovery of 78% of the total AM population in Fischer-344 rats. Lungs were removed and lavaged 20 times using 5 mL PBS each. Because our lavage technique was not as exhaustive as that derived by Lehnert and co-workers, it likely did not reach the entire epithelial surface of the lungs. Therefore, we attempted to apply their technique to estimate the AM population in our WKY rats. According to this protocol we obtained 9.76 ± 0.61 10^6^ AMs in three untreated adult healthy WKY rats. Assuming the same mean AM recovery efficiency of 78% Lehnert and co-workers observed, the corresponding total AM population was 12.5 ± 0.8 10^6^ AMs in our WKY rats.

### Estimated NP fraction on the epithelial surface

**Figure 4:**
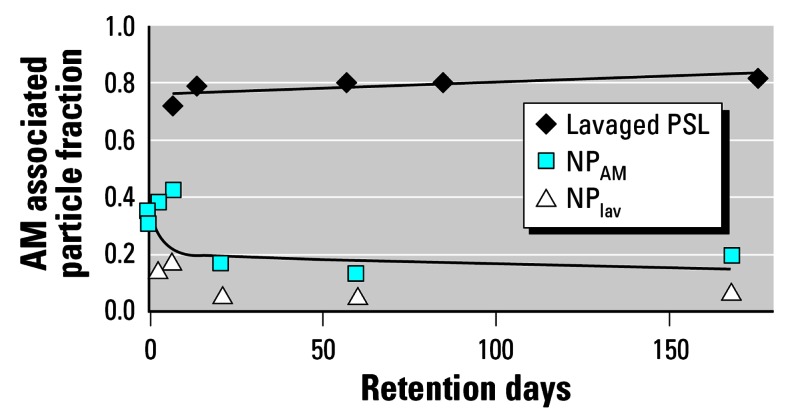
NP_lav_ and estimated NP fraction retained in the NP_AM_ together with biexponential fit: half-lives 2 and 800 days. In comparison, lavaged PSL fraction (2.1-μm polystyrene particles) in AMs are plotted together with a trend line as reported by Lehnert et al. (1989). All lavaged particle fractions are normalized to contemporary particle burden in the lungs.

To conservatively estimate the NP fraction associated with the entire AM population (NP_AM_) on the epithelial surface, we multiplied the NP fraction observed in the cell pellet by the ratio of the total AM population of 12.5 × 10^6^ AM over the actually lavaged AM fraction at each time point given in Table 1. Estimated NP_AM_ is shown in Figure 4. Although about 0.35 of the retained NPs was associated with AMs during the first week after inhalation, this fraction decreased slightly to about 0.2 during the time remaining. This is in clear contrast to the lavaged polystyrene particle (PSL) fractions in AMs, with a range of 0.8 of the lung burden as determined by Lehnert et al. (1989; Figure 4). In a comparable study on Syrian hamsters, Ellender et al. (1992) obtained fractions in BAL similar to those in the Lehnert study after the inhalation of glassy micron-sized glass particles.

**Figure 5:**
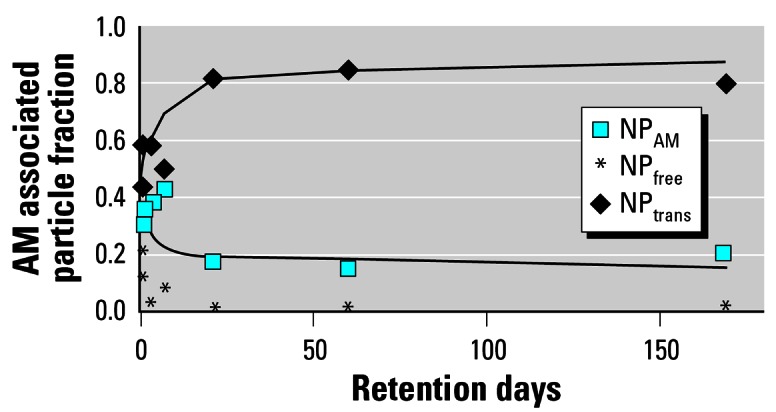
Estimated fraction of translocated NP into epithelium and interstitium as *NP_reloc_* = 1−(*NP_free_* + *NP_AM_*) with biexponential fit (half-lives 5 and 400 days) and the estimated fraction of NP_free_. NP_reloc_, relocated fraction. For comparison, the NP_AM_ data and the fitted curve of the AM-associated NP of Figure 4 are given. All NP fractions are normalized to contemporary particle burden in the lungs.

To estimate the total free NP fraction (NP_free_) on the epithelium, we performed the same type of estimate as for the NP_AM_, while basically correcting for the incomplete lavage procedure of the epithelial surface. Because the observed free NP fractions are very small 1 week after inhalation and later, the corrected fractions are still rather small and contribute little to the NP fraction on the epithelial surface. Estimated NP_free_ on the epithelium are shown in Figure 5.

### Relocation of NPs into and through the epithelium

Immediately after inhalation we were able to lavage free NPs from the epithelial surface (Table 1); of the lavageable NP fraction, 0.78 were free NPs. However, at later time points we found only negligible fractions of free NPs in BAL supernatant, clearly indicating that almost all NPs were associated either with AMs or with ECs, or were in the interstitial compartment. In addition, the lavageable NP_CP_ fraction associated with AMs declined rapidly so that the NP fraction retained in the lungs after lavage was about 0.85 of the contemporary lung burden during the first 3 weeks after inhalation; later it was higher than 0.90 (Table 1). Our conservative estimates to correct for either free or AM-associated NPs only slightly increased these fractions.

Although we principally cannot exclude that NPs stayed on the epithelium being firmly bound to ECs and were not accessible to lavage, it appears unlikely that the NPs continue to remain attached on the epithelium for as long as 6 months. In fact, NP binding to ECs should occur very rapidly, within seconds to minutes after deposition. This was not observed immediately after the 1-hr inhalation, at which time we were able to lavage a large fraction of free NPs. Therefore, we conclude that nonlavageable NP fractions retained longer than 3 days in the lungs were either internalized in ECs or had already been relocated into interstitial spaces. Furthermore, only negligible NP fractions were translocated to secondary target organs probably via blood circulation. Previously we have shown that this translocated NP fraction was < 1% of the deposited particles (Semmler et al. 2004). This relocated fraction (NP_reloc_) derived from *NP_reloc_* = 1−(*NP_free_* + *NP_AM_*) is shown in Figure 5.

These results are qualitatively confirmed by a number of morphologic studies: Takenaka et al. (2006) showed uptake in type I epithelial cells, endothelial cells, and the alveolar septum of ultrafine gold-particles by transmission electron microscopy, respectively, but found very few NPs on the epithelium. Furthermore, Ferin and co-workers had shown the uptake of 20-nm ultrafine TiO_2_-particles in epithelial cells and in the interstitial spaces (Ferin et al. 1992; Oberdörster et al. 1994). Geiser et al. (2005) showed that 1 hr after inhalation, 24% of inhaled 22-nm TiO_2_ particles were within or beyond the epithelial barrier of the lungs in epithelial and endothelial cells, connective tissue, or capillaries. Collectively, these studies showed that NPs penetrated into and beyond the epithelium rather rapidly. Furthermore, several publications have reported translocation into systemic circulation and accumulation in secondary target organs; these publications have been reviewed recently (Oberdörster et al. 2005). This is different from epithelial surface retention observed for micron-sized poly-styrene and glass particles (Lehnert et al. 1989; Oberdörster et al. 1994). Our data do not provide any information about the interstitial localization of retained NPs. Ferin and Oberdörster (1992) discussed the role of BALT as a possible site in rats where interstitial particles move out onto the bronchiolar mucosa. Green (1973) also suggested that in rodent lungs, coal-laden macrophages and particles exit onto airways in the same direction.

### Reentrainment of NPs from interstitium onto airways epithelium

As described previously (Semmler et al. 2004), long-term particle clearance of both ultrafine and micron-sized particles was dominated by macrophage-mediated particle transport from the peripheral lungs to the larynx, with subsequent passage through the GIT and fecal excretion. The kinetics of daily cleared particle fractions did not differ between ultrafine and micron-sized particles. The latter was demonstrated by the kinetics of fecal excretion rates (CRs) of poorly insoluble, micron-sized glass particles obtained from two strains of rats, Wistar-derived HMT rats and Fischer-344 rats (Semmler et al. 2004; Figure 4) as expressed by the equation:


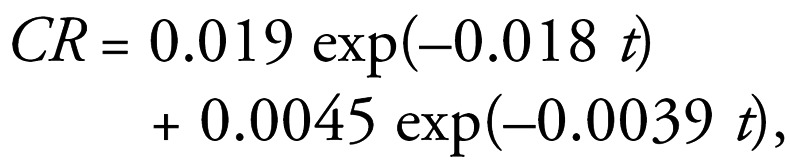


where *t* is time in days (Kreyling 1990).

**Figure 6:**
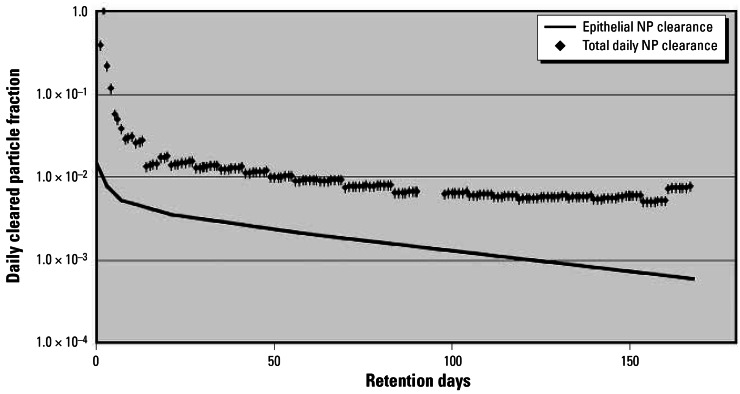
Estimated NP clearance from NP fraction retained on the epithelial surface versus observed daily fecal excretion as the result of AM-mediated NP transport from the lungs to larynx.

This result is indeed surprising, as NPs are predominantly translocated and retained beyond the epithelium, whereas most of the micron-sized PSLs were retained in AMs on the epithelium. If NP clearance had occurred only from the top of the epithelium, we can estimate this clearance kinetics from the above-calculated fractions of free and AM-associated Ir NPs and the clearance rates of the equation for CRs (Kreyling 1990). The curve is much lower than the observed excretion rates for Ir NPs shown in Figure 6. Therefore, the observed excretion rates representing macrophage-mediated NP transport from the peripheral lung epithelium to the larynx can be explained only by the fact that NPs—relocated and retained beyond the epithelial surface—needed to re-enter from the epithelium and interstitium onto the luminal side to get access to particle transport to the larynx as well as to BAL. This process of NP clearance from interstitial spaces and reentrainment on the epithelium appears to be continuous throughout the observed retention period as shown in Figure 6. The predominant fraction of phagocytized NPs at any long-term retention time point suggests that this reentrainment of the particles at the epithelial surface may be macrophage mediated; at least the very small fractions of free NPs in the BAL samples are suggesting this mechanism.

## Figures and Tables

**Table 1:** Cell counts and NP fractions in BAL fluid (mean ± SD; *n* = 4 WKY rats at each time point).^a^

Time after inhalation	Total no. of lavaged cells (× 10^6^)	PMNs (%)	NP_lav_^b^	NP_CP_^c^
Untreated healthy adult male WKY rats				
Control rats, nonexhaustive BAL	4.2 ± 0.45	0.7 ± 0.17		
Control rats, exhaustive BAL	9.76 ± 0.61	0.88 ± 1.13		
Clean air–exposed ventilated and intubated rats				
Control rats, exhaustive BAL	7.80 ± 0.28	3.67 ± 2.42		
1 day				
Ir NP–exposed ventilated and intubated rats				
0 hr	3.2 ± 0.1	4.05 ± 1.38	0.46 ± 0.05	0.22 ± 0.05
0.25 days	3.5 ± 1.2	6.41 ± 1.86	0.19 ± 0.03	0.62 ± 0.26
1 day	4.0 ± 1.0	5.88 ± 1.19	0.14 ± 0.03	0.71 ± 0.13
3 days	5.5 ± 1.4	1.25 ± 0.71	0.14 ± 0.03	0.92 ± 0.18
7 days	5.9 ± 1.1	0.55 ± 0.76	0.17 ± 0.04	0.84 ± 0.14
21 days	9.5 ± 3.7^d^	1.00 ± 0.71	0.06 ± 0.04	0.97 ± 0.66
60 days	1.8 ± 0.3	0.36 ± 0.48	0.05 ± 0.02	0.93 ± 0.28
168 days	2.4 ± 0.3	0.19 ± 0.24	0.07 ± 0.03	0.94 ± 0.38

Abbreviations: NP_CP_, nanoparticle fraction in the cell pellet of NP_lav_; NP_lav_, lavaged NP fraction of BAL; PMNs, polymorponuclear leukocytes.

a Controls for total cell numbers and PMNs were untreated.

b Data were normalized to the contemporary particle burden in the lungs (normalized to the deposited particle burden minus the already excreted fraction).

c Data were normalized to the totally lavaged particles in pellet and supernatant of BAL.

d Lavaged cell counts were very high without any indication for further health implications.
